# In vivo stable ^211^At-labeled prostate-specific membrane antigen-targeted tracer using a neopentyl glycol structure

**DOI:** 10.1186/s41181-024-00278-8

**Published:** 2024-06-17

**Authors:** Hiroyuki Suzuki, Kento Kannaka, Mizuki Hirayama, Tomoki Yamashita, Yuta Kaizuka, Ryota Kobayashi, Takahiro Yasuda, Kazuhiro Takahashi, Tomoya Uehara

**Affiliations:** 1 Graduate School of Pharmaceutical Sciences, Chiba University, 1-8-1 Chuo-Ku, Inohana, Chiba 260-8675 Japan; 2 Advanced Clinical Research Center, Fukushima Medical University, 1 Hikariga-Oka, Fukushima, 960-12195 Japan

**Keywords:** Astatine-211, PSMA, Neopentyl, Radiotheranostics

## Abstract

**Background:**

Prostate cancer is a common cancer among men worldwide that has a very poor prognosis, especially when it progresses to metastatic castration-resistant prostate cancer (mCRPC). Therefore, novel therapeutic agents for mCRPC are urgently required. Because prostate-specific membrane antigen (PSMA) is overexpressed in mCRPC, targeted alpha therapy (TAT) for PSMA is a promising treatment for mCRPC. Astatine-211 (^211^At) is a versatile α-emitting radionuclide that can be produced using a cyclotron. Therefore, ^211^At-labeled PSMA compounds could be useful for TAT; however, ^211^At-labeled compounds are unstable against deastatination in vivo. In this study, to develop in vivo stable ^211^At-labeled PSMA derivatives, we designed and synthesized ^211^At-labeled PSMA derivatives using a neopentyl glycol (NpG) structure that can stably retain ^211^At in vivo. We also evaluated their biodistribution in normal and tumor-bearing mice.

**Results:**

We designed and synthesized ^211^At-labeled PSMA derivatives containing two glutamic acid (Glu) linkers between the NpG structure and asymmetric urea (NpG-L-PSMA ((L-Glu)_2_ linker used) and NpG-D-PSMA ((D-Glu)_2_ linker used)). First, we evaluated the characteristics of ^125^I-labeled NpG derivatives because ^125^I was readily available. [^125^I]I-NpG-L-PSMA and [^125^I]I-NpG-D-PSMA showed low accumulation in the stomach and thyroid, indicating their high in vivo stability against deiodination. [^125^I]I-NpG-L-PSMA was excreted in urine as hydrophilic radiometabolites in addition to the intact form. Meanwhile, [^125^I]I-NpG-D-PSMA was excreted in urine in an intact form. In both cases, no radioactivity was observed in the free iodine fraction. [^125^I]I-NpG-D-PSMA showed higher tumor accumulation than [^125^I]I-NpG-L-PSMA. We then developed ^211^At-labeled PSMA using the NpG-D-PSMA structure. [^211^At]At-NpG-D-PSMA showed low accumulation in the stomach and thyroid in normal mice, indicating its high stability against deastatination in vivo. Moreover, [^211^At]At-NpG-D-PSMA showed high accumulation in tumor similar to that of [^125^I]I-NpG-D-PSMA.

**Conclusions:**

[^211^At]At-NpG-D-PSMA showed high in vivo stability against deastatination and high tumor accumulation. [^211^At]At-NpG-D-PSMA should be considered as a potential new TAT for mCRPC.

**Supplementary Information:**

The online version contains supplementary material available at 10.1186/s41181-024-00278-8.

## Background

**Fig. 1:**
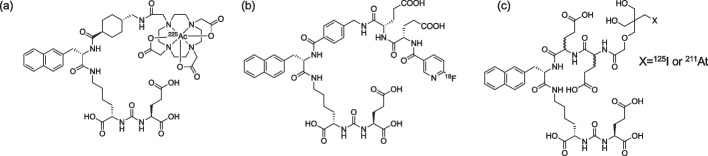
Structures of **a** [^225^Ac]Ac-PSMA-617, **b** [^18^F]F-PSMA-1007, and **c** [X]X-NpG-D/L-PSMA. (X = ^125^I, ^211^At)

Prostate cancer is the second most common cancer among men worldwide (Sung et al. 2021). Prostate cancer, especially when it progresses to metastatic castration-resistant prostate cancer (mCRPC), which is resistant to standard care such as hormonal therapy or chemotherapy, has a very poor prognosis (Chandrasekar et al. 2015; Kirby et al. 2011). Therefore, novel therapeutic agents for mCRPC are urgently required. Prostate-specific membrane antigen (PSMA) is a type II membrane glycoprotein that is overexpressed in prostate cancer (Silver et al. 1997). Because PSMA has been recognized as a reliable biomarker that reflects the disease burden of differentiated prostate cancer and mCRPC, it is a promising target for mCRPC (Bouchelouche et al. 2010). Radionuclide therapy using α-emitting radionuclides and β-emitting radionuclides that target PSMA has been widely investigated to treat mCRPC more efficiently (Jones et al. 2020). Among these therapies, radionuclide therapy using α-emitting radionuclides, known as targeted alpha therapy (TAT), is expected to have high therapeutic efficacy and low side effects owing to the higher energy transfer rates and shorter pathlengths of α-emitting radionuclides compared to other radionuclides (Nelson et al. 2021; Bruland et al. 2023). Indeed, an actinium-225 (^225^Ac)-labeled compound targeting PSMA, developed as a TAT agent for mCRPC ([^225^Ac]Ac-PSMA-617, Fig. 1a), has attracted much attention because it leads to complete remission in patients with late-stage mCRPC (Kratochwil et al. 2016). However, the supply of ^225^Ac is very limited, and production methods are currently under development. ^225^Ac generates multiple α-emitting daughter radionuclides, but the nuclear recoil energy can release daughter radionuclides from the labeled compound, leading to unintended irradiation of non-target tissues. Therefore, new versatile TAT agents using α-emitting radionuclides are expected to be developed (Nelson et al. 2021; Nagatsu et al. 2021).

Astatine-211 (^211^At) is an easy-to-handle α-emitting radionuclide with a half-life of 7.2 h. It is a versatile α-emitting radionuclide that can be produced using a 30 MeV cyclotron (Nelson et al. 2021; Zalutsky and Pruszynski 2011). Furthermore, because ^211^At emits only one alpha particle during decay, the potential radiotoxicity of its daughter radionuclides is negligible. As such, ^211^At-labeled PSMA derivatives are expected to be versatile TAT compounds (Chakravarty et al. 2023). In general, conventional ^211^At-labeled compounds that use astatobenzene structures as ^211^At-labeling moieties have the problem that ^211^At is released in vivo (Hadley et al. 1991; Watabe et al. 2023; Kiess et al. 2016). In particular, low molecular weight compounds labeled with ^211^At using astatobenzene structure were less stable against deastatination in vivo. This has limited the development of ^211^At-labeled PSMA derivatives using astatobenzene structure as the ^211^At-labeling moiety. On the other hand, metabolically stabile cyclic peptides such as the RGD peptide labeled with ^211^At using astatobenzene showed high in vivo stability (Echigo et al. 2024; Ogawa et al. 2021). These results suggest that deastatination is involved in enzyme recognition and that astatobenzene derivatives, which are less easily recognized by enzymes, are stable in vivo. Recently, in vivo stable ^211^At-labeled PSMA derivatives using astatobenzene structures have been developed that have high tumor growth suppression and low side effects in normal tissues (Mease et al. 2022; Fakiri et al. 2024). In these compounds, astatobenzene structure was placed in the middle of the compounds. The insertion in this position may have prevented it from being recognized by enzymes involved in deastatination. Recently, we developed a neopentyl derivative with two hydroxy groups (NpG structure) as a ^211^At-labeling moiety that could stably retain ^211^At in vivo (Suzuki et al. 2021; Kaizuka et al. 2024) even when a low-molecular-weight compound was used as a targeting moiety. Therefore, it is thought that NpG structure could be used to design a variety of ^211^At-labeled PSMA derivatives.

In this study, we designed and synthesized ^211^At-labeled PSMA derivatives that contained two glutamic acid (Glu) linkers between the NpG structure and the PSMA binding moiety, asymmetric urea (NpG-L-PSMA ((L-Glu)_2_ linker used) and NpG-D-PSMA ((D-Glu)_2_ linker used)) (Fig. 1c). First, we evaluated the characteristics of ^125^I-labeled NpG derivatives because ^125^I was readily available. Then, the biodistribution of ^211^At-labeled NpG derivatives in mice was evaluated.

## Methods

The preparation of [^211^At]At-NpG-D-PSMA, [^125^I]I-NpG-D-PSMA, and [^125^I]I-NpG-L-PSMA and the analytical methods using reversed-phase high-performance liquid chromatography (RP-HPLC) were described in the Supporting Information.

### Cell line and in vitro assay

The human prostate cancer cell line LNCaP was provided by RIKEN BioResource Center (BRC) through the National BioResource Project of MEXT/AMED, Japan. LNCaP cells were cultured according to protocols prescribed by RIKEN BRC. LNCaP cells were seeded in 24-well plates (~ 1.5 × 10^5^ cells/500 µL/well) coated with 0.01% poly-L-lysine (Sigma-Aldrich, Tokyo). After washing twice with assay medium (RPMI 1640 supplemented with 0.5% BSA), 250 µL of assay medium was added to each well. Each PSMA derivative and [^125^I]DCIT dissolved in assay medium (250 µL) were added to the well, and the plates were then incubated at 37 °C for 1 h. After removing the assay medium, the cells were washed twice with assay medium (250 µL × 2). All cells were lysed with 1 M NaOH (500 µL), and the solution was collected. The cells were washed twice with PBS (250 µL × 2), and the solution was mixed with the previous solution. The radioactivity in the solution was measured using an auto-well gamma counter. The IC_50_ value was calculated by fitting with nonlinear regression using GraphPad Prism 8.4.3 (GraphPad Software, Inc., San Diego).

### Preparation of the animals

Animal studies were conducted in accordance with the institutional guidelines approved by the Chiba University Animal Care Committee (Permit No. 4–183). Five-week-old male ddY mice and BALB/c-nu/nu mice were purchased from Japan SLC Inc. (Hamamatsu, Japan). LNCaP cells (5.0 × 10^6^ cells) were suspended in 100 μL of PBS and Matrigel (BD Biosciences, Franklin Lakes, NJ, USA) in a 1:1 ratio. Tumor xenograft models were prepared via subcutaneous injection of this LNCaP cell suspension into BALB/c nude mice.

### In vivo study

Six-week-old male ddY mice were injected via the tail vein with each radiolabeled PSMA derivative. Animals were sacrificed and the organs were dissected at 10 min, 1 h, 3 h, and 6 h after injection. Metabolic cages were used for housing normal mice. The tissues of interest were excised and weighed, and the radioactivity counts in each tissue were determined using an auto-well gamma counter. Each value was expressed as the mean percent injected dose/g tissue ± SD for a group of 3–5 animals, except those for the stomach, intestine, and thyroid.

Biodistribution studies were conducted using male BALB/c-nu/nu mice bearing LNCaP cell xenografts at 1 and 3 h postinjection of [^211^At]At-NpG-D-PSMA (100 µL, 37 kBq) and 1 and 6 h postinjection of ^125^I-labeled PSMA derivative (100 µL, 11.1 kBq). In the blocking study, [^125^I]I-NpG-D-PSMA was coinjected with 2-PMPA (50 mg/kg weight) into tumor-bearing mice via the tail vein. At 1 h postinjection, the mice were sacrificed. The tissues of interest were excised and weighed, and the radioactivity counts in each tissue were determined using an auto-well gamma counter. Each value was expressed as the mean percent injected dose/g tissue ± SD for a group of 3–5 animals, except those for the stomach, intestine, and thyroid.

### Analysis of radiometabolites in urine

Urine samples were collected from 6-week-old ddY mice 6 h after injection of each ^125^I-labeled PSMA derivative (100 µL, 370 kBq). After ethanol precipitation of proteins, the urine samples were filtered through a polycarbonate membrane (0.45 µm) and analyzed by RP-HPLC (System 2).

### Statical analysis

All data were presented as the mean ± standard deviation (SD) of at least three independent measurements. The results of the biodistribution studies were analyzed using Student’s *t*-test or one-way analysis of variance followed by Tukey’s test for multiple comparisons (GraphPad Prism 8.4.3). Significance was assigned at *p* < 0.05.

## Results

### Synthesis and radiolabeling

**Scheme 1:**
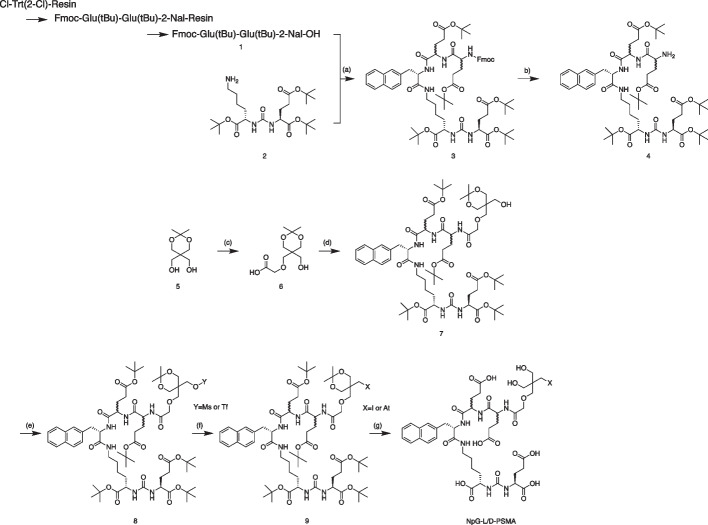
Synthetic scheme of NpG-D/L-PSMA. **a** HOBt, DIPEA, WSCI/HCl, **b** 20% piperidine/DMF, **c** NaH, bromoacetic acid, **d** HOBt, DIPEA, WSCI/HCl, **e** 2,6-lutidine, trifluoromethanesulfonic anhydride or methanesulfonyl chloride; **f** NaI, [^125^I]NaI, or [^211^At]At, **g** TFA/H_2_O = 9/1

NpG-D-PSMA and NpG-L-PSMA were synthesized in a similar manner (Scheme 1). The tripeptide spacer (compound **1**) linking the asymmetric urea structure, compound **2** (PSMA binding site), to the NpG structure (radiohalogen labeling site) was synthesized by a solid-phase method using Cl-Trt(2-Cl) resin. Compound **3** was obtained by condensation of compound **1**, with the asymmetric urea structure synthesized according to a previous report (Maresca et al. 2009), and compound **4** was obtained by deprotection of the Fmoc group of compound **3**. The ^211^At/^125^I-labeling moiety, the NpG structure, was synthesized using pentaerythritol as the starting material. Compound **6** was obtained by introducing a carboxyl group into one of the remaining hydroxy groups using bromoacetic acid. Compound **7** was obtained from condensation of compound **6** with compound **4**. The hydroxy group of compound **7** was activated with a triflate or methylate group to obtain the labeling precursor (compound **8**). The radiochemical yields of the ^125^I-labeled and ^211^At-labeled PSMA derivatives (two steps) were 19% ([^125^I]I-NpG-L-PSMA), 25% ([^125^I]I-NpG-D-PSMA), and 22% ([^211^At]At-NpG-D-PSMA), respectively, and the radiochemical purity of all radiolabeled PSMA derivatives was over 95%. The retention times of [^125^I]I-NpG-L-PSMA and [^125^I]I-NpG-D-PSMA were identical to those of their non-radioactive iodine-labeled counterparts. Because there is no stable isotope for astatine, [^211^At]At-NpG-D-PSMA was confirmed by the similarity of the retention time during RP-HPLC with that of its non-radioactive iodine-labeled counterpart, I-NpG-D-PSMA (Fig. S1).

### Lipophilicity

The log *D*_7.4_ value of [^125^I]I-NpG-D-PSMA was − 3.14 ± 0.03, which was lower than that of [^18^F]F-PSMA-1007 (− 1.6) (Robu et al. 2018) and higher than that of [^68^Ga]Ga-PSMA-617 (− 4.30 ± 0.10) (Umbricht et al. 2017).

### Binding affinity

The binding affinity of I-NpG-L-PSMA and I-NpG-D-PSMA to LNCaP cells was evaluated by calculating the IC_50_ by competitive inhibition experiments using the binding of [^125^I]DCIT to LNCaP cells as a competitor. Ga-labeled PSMA-617 was used as a reference compound (Table 1, Fig. S2). The IC_50_ values of I-NpG-L-PSMA, I-NpG-D-PSMA, and Ga-PSMA-617 were similar.

**Table 1:** The IC_50_ values of each compound

Compound	IC_50_, nM	95% C.I.^a^
I-NpG-L-PSMA	4.04	3.37–5.85
I-NpG-D-PSMA	6.06	5.26–6.98
Ga-PSMA-617	7.56	6.57–8.78

^a^C.I. = confidential interval

### Biodistribution in normal mice

**Fig. 2:**
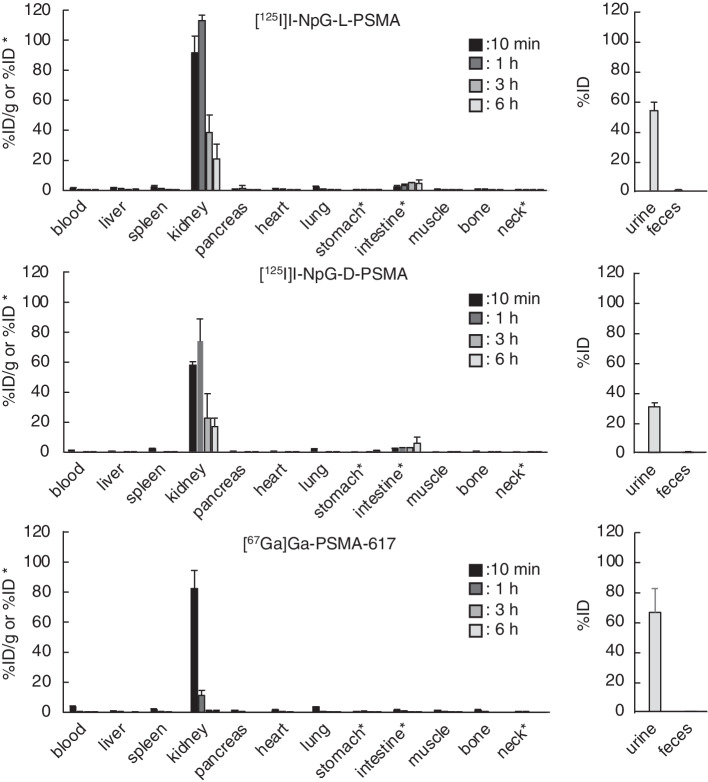
Radioactivity in the tissues after 10 min, 1 h, 3 h, and 6 h injection of ^125^I-labeled compounds and ^67^Ga-labeled compound in normal mice

Figure 2 and Table S1 show the biodistribution of radioactivity after injection of [^125^I]I-NpG-L-PSMA and [^125^I]I-NpG-D-PSMA in normal mice. [^125^I]I-NpG-L-PSMA and [^125^I]I-NpG-D-PSMA showed low accumulation in the stomach and thyroid, indicating high stability against deiodination in the body. [^125^I]I-NpG-D-PSMA showed biodistribution similar to that of [^125^I]I-NpG-L-PSMA in normal mice. Moreover, both ^125^I-labeled PSMA derivatives showed similar biodistribution to that of [^67^Ga]Ga-PSMA-617 in normal mice, except for in the kidney and intestine.

### Urine analysis

**Fig. 3:**
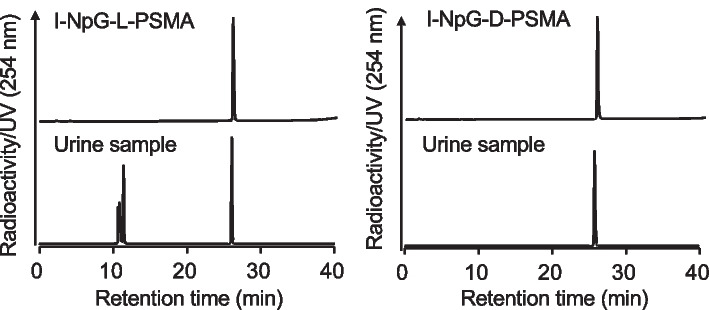
RP-HPLC analysis of urine samples after injection of [^125^I]I-NpG-L-PSMA (left) and [^125^I]I-NpG-D-PSMA (right)

[^125^I]I-NpG-L-PSMA and [^125^I]I-NpG-D-PSMA were injected into normal mice, and the radioactivity excreted in urine up to 6 h after injection was analyzed by RP-HPLC (Fig. 3). In the case of [^125^I]I-NpG-L-PSMA, radioactivity was excreted as intact [^125^I]I-NpG-L-PSMA and as radiometabolites with shorter retention times than [^125^I]I-NpG-L-PSMA, indicating that radiometabolites were more hydrophilic than [^125^I]I-NpG-L-PSMA. In contrast, in the case of [^125^I]I-NpG-D-PSMA, radioactivity was excreted as only intact [^125^I]I-NpG-D-PSMA. In both cases, there was no radioactivity in the free iodine fraction.

### Biodistribution in tumor-bearing mice

**Fig. 4:**
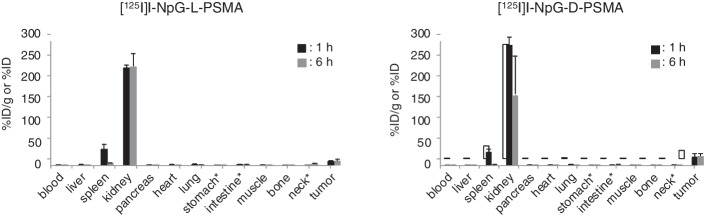
Radioactivity in the tissues after 1 h and 6 h injection of [^125^I]I-NpG-L-PSMA and [^125^I]I-NpG-D-PSMA in tumor bearing mice

**Fig. 5:**
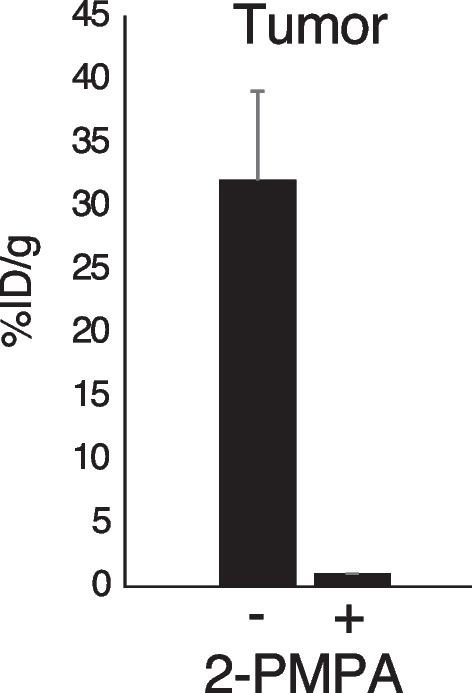
Tumor accumulation of [^125^I]I-NpG-D-PSMA with or without 2-PMPA

Figure 4 and Table S2 show the biodistribution of radioactivity after injection of [^125^I]I-NpG-L-PSMA and [^125^I]I-NpG-D-PSMA in tumor-bearing mice. [^125^I]I-NpG-D-PSMA showed similar biodistribution in tumor-bearing mice to [^125^I]I-NpG-L-PSMA, as expected for tumors. The tumor accumulation of [^125^I]I-NpG-D-PSMA was higher than that of [^125^I]I-NpG-L-PSMA. In addition, accumulation of [^125^I]I-NpG-D-PSMA in the tumor and spleen was inhibited by 2-PMPA, a PSMA inhibitor, suggesting that [^125^I]I-NpG-D-PSMA specifically bound to PSMA (Fig. 5). Next, we prepared [^211^At]At-NpG-D-PSMA and then evaluated the biodistribution of radioactivity in tumor-bearing mice after injection of [^211^At]At-NpG-D-PSMA. [^211^At]At-NpG-D-PSMA showed low accumulation in the stomach and thyroid, indicating that it was stable against deastatination in vivo. Moreover, [^211^At]At-NpG-D-PSMA showed high accumulation in the tumor, similar to that of [^125^I]I-NpG-D-PSMA (Table 2).

**Table 2:** Biodistribution of radioactivity in tumor-bearing mice after injection of [^211^At]At-NpG-D-PSMA

Tissue radioactivity is expressed as %ID/g [for each group, n = 3; results are reported as mean ± SD.]		
Tissues	1 h	3 h
Blood	0.41 ± 0.10	0.09 ± 0.02
Liver	0.38 ± 0.08	0.11 ± 0.04
Spleen	11.8 ± 1.26	4.67 ± 3.27
Kidney	181 ± 13.5	132 ± 37.8
Pancreas	0.95 ± 0.48	0.27 ± 0.05
Heart	0.65 ± 0.22	0.23 ± 0.08
Lung	1.84 ± 0.22	0.55 ± 0.06
Muscle	0.30 ± 0.12	0.13 ± 0.09
Bone	0.40 ± 0.22	0.29 ± 0.18
Intestine*	1.94 ± 0.22	1.74 ± 0.39
Stomach*	0.37 ± 0.15	0.55 ± 0.33
Neck*	0.04 ± 0.03	0.05 ± 0.04
Tumor	13.4 ± 3.17	16.9 ± 8.45

^*^Tissue radioactivity was expressed as %ID

## Discussion

In this study, we designed and synthesized ^211^At-labeled PSMA derivatives using an NpG structure that is stable against dehalogenation in vivo (Suzuki et al. 2021). Initially, we designed and synthesized NpG-conjugated PSMA without a glutamic acid linker (NpG-PSMA, Fig. S3), but this compound showed high accumulation in the liver and intestine and low accumulation in the kidney, which was not observed with common PSMA derivatives (Cardinale et al. 2017) (Table S3). The lipophilicity of the NpG structure was considered to be higher than that of the DOTA metal complex. Based on these findings, new NpG-conjugated PSMA derivatives containing two glutamic acids as a hydrophilic linker, similar to the structure of [^18^F]F-PSMA-1007 (Fig. 1), were designed and synthesized to increase the hydrophilicity of the PSMA derivatives. The log *D*_7.4_ value of [^125^I]I-NpG-D-PSMA was -3.14 ± 0.03, which was lower than that of [^18^F]F-PSMA-1007 (-1.6) (Robu et al. 2018). The difference in the log *D*_7.4_ value of each compound may be due to differences in the lipophilicity of the radiolabeling moiety such as NpG structure and pyridine structure. Although the log *D*_7.4_ value of [^125^I]I-NpG-D-PSMA was higher than that of [^68^Ga]Ga-PSMA-617, this value was considered sufficient to improve the biodistribution of radiolabeled PSMA derivative.

[^18^F]F-PSMA-1007 contains two L-form glutamic acids as linkers in its structure (Fig. 1b) (Cardinale et al. 2017). In addition, [^123^I]IGLCE also contains L-form amino acids (Fig. S4) (Harada et al. 2013). Therefore, we first designed and synthesized NpG-L-PSMA containing L-form glutamic acids as linkers. I-NpG-L-PSMA showed similar binding affinity to LNCaP cells as Ga-PSMA-617 (Table 2), indicating that structural modifications away from the asymmetric urea structure, the PSMA binding site, would not affect the binding affinity to PSMA. [^125^I]I-NpG-L-PSMA showed rapid blood clearance and high accumulation in the kidney (Fig. 2). This biodistribution pattern is similar to that of common radiolabeled PSMA derivatives (Cardinale et al. 2017). Compared with [^67^Ga]Ga-PSMA-617, [^125^I]I-NpG-L-PSMA showed slower kidney clearance (Fig. 2, Table S1). [^18^F]F-PSMA-1007, which had a structure similar to that of [^125^I]I-NpG-L-PSMA, also showed slow kidney clearance (Cardinale et al. 2017). These results suggest that the glutamic acid linker may be involved in renal accumulation.

When urine samples were analyzed by RP-HPLC after injection of [^125^I]I-NpG-L-PSMA, radiometabolites were observed (Fig. 3). Similar results have been reported for [^68^Ga]GaDOTAGA-FFK(Sub-KuE), which contains an L-form amino acid sequence (Weineisen et al. 2014). This result suggests that the L-form glutamic acid linker in [^125^I]I-NpG-L-PSMA was metabolized in the body. Although the structures of radiometabolites were not investigated, [^125^I]I-NpG-D-PSMA showed high in vivo stability (Fig. 3), suggesting that the L-glutamic acid linker was likely to be cleaved. [^125^I]I-NpG-D-PSMA showed similar binding affinity for LNCaP cells to that of [^125^I]I-NpG-L-PSMA. This finding was consistent with the finding that the binding affinity of GaDOTAGA-ffk(Sub-KuE), which contains D-form amino acids, was similar to that of GaDOTAGA-FFK(Sub-KuE), which contains L-form amino acids (Weineisen et al. 2014). These findings suggested that stereoisomers away from the asymmetric urea structure, the PSMA binding site, do not affect the binding affinity to PSMA. Meanwhile, tumor accumulation of [^125^I]I-NpG-D-PSMA was higher than that of [^125^I]I-NpG-L-PSMA (Fig. 4, Table S2). This finding is consistent that of a previous study, where it was found that tumor accumulation of GaDOTAGA-ffk(Sub-KuE), containing D-form amino acids, was higher than that of GaDOTAGA-FFK(Sub-KuE), containing L-form amino acids (Weineisen et al. 2014). These results suggest that the in vivo stability of radiolabeled PSMA derivatives affects tumor accumulation in vivo. Furthermore, the kidney retention of [^125^I]I-NpG-D-PSMA was shorter than that of [^125^I]I-NpG-L-PSMA (Figs. 2, 4 and Tables S1, S2). The previously reported ^211^At-labeled PSMA derivatives containing the D-form glutamic acid linker also showed shorter retention times than those containing the L-form glutamic acid linker (Watabe et al. 2023). These results indicate that the glutamic acid linker affects renal radioactivity behavior. On the other hand, [^211^At]At-NpG-D-PSMA showed higher accumulation in the kidney than the compound, ^211^At-3-Lu, reported by Mease (Mease et al. 2022) and was similar to the compound, [^211^At]-PSAt-3, reported by Fakiri (Fakiri et al. 2024). The structures of ^211^At-3-Lu and [^211^At]-PSAt-3 were similar except for the linker structure. These results suggested that the linker structure would affect the renal accumulation of radiolabeled PSMA derivatives although the mechanism was not clear. Although [^211^At]At-NpG-D-PSMA showed higher accumulation in the kidney, the values were comparable to those of common radiolabeled PSMA derivatives and not considered problematic.

Because the [^125^I]I-NpG-D-PSMA structure that contained D-form glutamic acids showed high tumor accumulation and rapid clearance from the kidney compared to [^125^I]I-NpG-L-PSMA, we developed a radiolabeled compound in which the ^125^I of [^125^I]I-NpG-D-PSMA was replaced with ^211^At. [^211^At]At-NpG-D-PSMA, like [^125^I]I-NpG-D-PSMA, showed low accumulation in the stomach and thyroid in tumor-bearing mice (Tables 2, S2), indicating that the NpG structure could hold ^211^At stably in vivo even when bound to PSMA derivatives. Radioactivity levels in the tumor after injection of [^211^At]At-NpG-D-PSMA were maintained from 1 to 3 h, whereas the renal radioactivity levels of [^211^At]At-NpG-D-PSMA decreased from 1 to 3 h, similar to those of [^125^I]I-NpG-D-PSMA. These results suggest that [^211^At]At-NpG-D-PSMA is a promising TAT agent against mCRPC. Because of the limited availability of ^211^At at our facility, we could not perform the therapeutic study using [^211^At]At-NpG-D-PSMA. The similar biodistribution of [^211^At]At-NpG-D-PSMA and [^125^I]I-NpG-D-PSMA suggested that radiotheranostics using [^123/131^I]I-NpG-D-PSMA and [^211^At]At-NpG-D-PSMA could be useful. In addition, because the NpG structure could be used as an ^18^F-labeling moiety (Tago et al. 2021; Shimizu et al. 2019), similar results can be expected for radiotheranostics using [^18^F]F-NpG-D-PSMA and [^211^At]At-NpG-D-PSMA.

## Conclusions

[^211^At]At-NpG-D-PSMA was synthesized by incorporating an NpG structure into the asymmetric urea structure via a D-glutamic acid linker. [^211^At]At-NpG-D-PSMA showed good stability in the body and high accumulation in tumors. These findings suggest that [^211^At]At-NpG-D-PSMA can be used as a potential new TAT agent for mCRPC.

### Supplementary Information

Additional file 1.

## Data Availability

Data and materials are available from the corresponding authors upon reasonable request.

## Abbreviations

mCRPC: Metastatic castration-resistant prostate cancer
PSMA: Prostate-specific membrane antigen
TAT: Targeted alpha-therapy
NpG: Neopentyl derivative with two hydroxy groups, neopentyl glycol
RP-HPLC: Reversed phase- high performance liquid chromatography
[^125^I]DCIT: *N*-(*N*-((*S*)-1,3-Dicarobxypropyl)carbamoyl)-*S*-3-iodo-L-tyrosine
[^123^I]IGLCE: (2S)-2-(3-((1R)-1-Carboxy-2-((1-((R)-5-carboxy-5-(2-(3-iodobenzamido)acetamido)pentyl)-2,5-dioxopyrrolidin-3-yl)- thio)ethyl)ureido)pentanedioic Acid
2-PMPA: 2-(Phosphonomethyl)pentanedioic acid

## References

[CR1] Bouchelouche K, Choyke PL, Capala J (2010). Prostate specific membrane antigen- a target for imaging and therapy with radionuclides. Discov Med.

[CR2] Bruland OS, Larsen RH, Baum RP, Juzeniene A (2023). Editorial: targeted alpha particle therapy in oncology. Front Med (lausanne).

[CR3] Cardinale J, Schafer M, Benesova M, Bauder-Wust U, Leotta K, Eder M (2017). Preclinical evaluation of ^18^F-PSMA-1007, a new prostate-specific membrane antigen ligand for prostate cancer imaging. J Nucl Med.

[CR4] Chakravarty R, Lan X, Chakraborty S, Cai W (2023). Astatine-211 for PSMA-targeted alpha-radiation therapy of micrometastatic prostate cancer: a sustainable approach towards precision oncology. Eur J Nucl Med Mol Imaging.

[CR5] Chandrasekar T, Yang JC, Gao AC, Evans CP (2015). Mechanisms of resistance in castration-resistant prostate cancer (CRPC). Transl Androl Urol.

[CR6] Echigo H, Mishiro K, Munekane M, Fuchigami T, Washiyama K, Takahashi K (2024). Development of probes for radiotheranostics with albumin binding moiety to increase the therapeutic effects of astatine-211 (^211^At). Eur J Nucl Med Mol Imaging.

[CR7] El Fakiri M, Ayada N, Muller M, Hvass L, Gamzov TH, Clausen AS (2024). Development and preclinical evaluation of [^211^At]PSAt-3-Ga: an inhibitor for targeted alpha-therapy of prostate cancer. J Nucl Med.

[CR8] Hadley SW, Wilbur DS, Gray MA, Atcher RW (1991). Astatine-211 labeling of an antimelanoma antibody and its fab fragment using N-succinimidyl p-astatobenzoate: comparisons *in vivo* with the p-[^125^I]iodobenzoyl conjugate. Bioconjugate Chem.

[CR9] Harada N, Kimura H, Ono M, Saji H (2013). Preparation of asymmetric urea derivatives that target prostate-specific membrane antigen for SPECT imaging. J Med Chem.

[CR10] Jones W, Griffiths K, Barata PC, Paller CJ (2020). PSMA theranostics: review of the current status of PSMA-targeted imaging and radioligand therapy. Cancers (basel)..

[CR11] Kaizuka Y, Suzuki H, Watabe T, Ooe K, Toyoshima A, Takahashi K (2024). Neopentyl glycol-based radiohalogen-labeled amino acid derivatives for cancer radiotheranostics. EJNMMI Radiopharm Chem.

[CR12] Kiess AP, Minn I, Vaidyanathan G, Hobbs RF, Josefsson A, Shen C (2016). (2S)-2-(3-(1-Carboxy-5-(4–^211^At-Astatobenzamido)Pentyl)Ureido)-pentanedioic acid for PSMA-targeted alpha-particle radiopharmaceutical therapy. J Nucl Med.

[CR13] Kirby M, Hirst C, Crawford ED (2011). Characterising the castration-resistant prostate cancer population: a systematic review. Int J Clin Pract.

[CR14] Kratochwil C, Bruchertseifer F, Giesel FL, Weis M, Verburg FA, Mottaghy F (2016). ^225^Ac-PSMA-617 for PSMA-targeted alpha-radiation therapy of metastatic castration-resistant prostate cancer. J Nucl Med.

[CR15] Maresca KP, Hillier SM, Femia FJ, Keith D, Barone C, Joyal JL (2009). A series of halogenated heterodimeric inhibitors of prostate specific membrane antigen (PSMA) as radiolabeled probes for targeting prostate cancer. J Med Chem.

[CR16] Mease RC, Kang CM, Kumar V, Banerjee SR, Minn I, Brummet M (2022). An improved ^211^At-Labeled agent for PSMA-targeted alpha-therapy. J Nucl Med.

[CR17] Nagatsu K, Suzuki H, Fukada M, Ito T, Ichinose J, Honda Y (2021). Cyclotron production of ^225^Ac from an electroplated ^226^Ra target. Eur J Nucl Med Mol Imaging.

[CR18] Nelson BJ, Andersson JD, Wuest F (2021). Targeted alpha therapy: progress in radionuclide produciton, radiochemistry, and applications. Pharmaceuticals (basel).

[CR19] Ogawa K, Echigo H, Mishiro K, Hirata S, Washiyama K, Kitamura Y (2021). ^68^Ga- and ^211^At-Labeled RGD peptides for radiotheranostics with multiradionuclides. Mol Pharm.

[CR20] Robu S, Schmidt A, Eiber M, Schottelius M, Gunther T, Hooshyar Yousefi B (2018). Synthesis and preclinical evaluation of novel ^18^F-labeled Glu-urea-Glu-based PSMA inhibitors for prostate cancer imaging: a comparison with ^18^F-DCFPyl and ^18^F-PSMA-1007. EJNMMI Res.

[CR21] Shimizu Y, Zhao S, Yasui H, Nishijima KI, Matsumoto H, Shiga T (2019). A novel PET probe "[^18^F]DiFA" accumulates in hypoxic region *via* glutathione conjugation following reductive metabolism. Mol Imaging Biol.

[CR22] Silver DA, Pellicer I, Fair WR, Heston WD, Cordon-Cardo C (1997). Prostate-specific membrane antigen expression in normal and malignant human tissues. Clin Cancer Res.

[CR23] Sung H, Ferlay J, Siegel RL, Laversanne M, Soerjomataram I, Jemal A (2021). Global cancer statistics 2020: GLOBOCAN estimates of incidence and mortality worldwide for 36 cancers in 185 countries. CA Cancer J Clin.

[CR24] Suzuki H, Kaizuka Y, Tatsuta M, Tanaka H, Washiya N, Shirakami Y (2021). Neopentyl glycol as a scaffold to provide radiohalogenated theranostic pairs of high *in vivo* stability. J Med Chem.

[CR25] Tago T, Toyohara J, Fujimaki R, Tatsuta M, Song R, Hirano K (2021). Effects of ^18^F-fluorinated neopentyl glycol side-chain on the biological characteristics of stilbene amyloid-beta PET ligands. Nucl Med Biol.

[CR26] Umbricht CA, Benesova M, Schmid RM, Turler A, Schibli R, van der Meulen NP (2017). ^44^Sc-PSMA-617 for radiotheragnostics in tandem with ^177^Lu-PSMA-617-preclinical investigations in comparison with ^68^Ga-PSMA-11 and ^68^Ga-PSMA-617. EJNMMI Res.

[CR27] Watabe T, Kaneda-Nakashima K, Shirakami Y, Kadonaga Y, Ooe K, Wang Y (2023). Targeted alpha-therapy using astatine (^211^At)-labeled PSMA1, 5, and 6: a preclinical evaluation as a novel compound. Eur J Nucl Med Mol Imaging.

[CR28] Weineisen M, Simecek J, Schottelius M, Schwaiger M, Wester HJ (2014). Synthesis and preclinical evaluation of DOTAGA-conjugated PSMA ligands for functional imaging and endoradiotherapy of prostate cancer. EJNMMI Res.

[CR29] Zalutsky MR, Pruszynski M (2011). Astatine-211: production and availability. Curr Radiopharm.

