# Supplementary material for: In vivo stable 211At-labeled prostate-specific membrane antigen-targeted tracer using a neopentyl glycol structure
Source: EJNMMI Radiopharm Chem. 2024 Jun 17;9:48. doi: 10.1186/s41181-024-00278-8 (PMC11183015; doi:10.1186/s41181-024-00278-8)
Supplement: Supplementary file 1 — Additional file 1. [file 41181_2024_278_MOESM1_ESM.docx]

**Supporting Information**

***In vivo* stable ^211^At-labeled prostate-specific membrane antigen-targeted tracer using a neopentyl glycol structure**

Hiroyuki Suzuki^1^, Kento Kannaka^1^, Mizuki Hirayama^1^, Tomoki Yamashita^1^, Yuta Kaizuka^1^, Ryota Kobayashi^1^, Takahiro Yasuda^1^, Kazuhiro Takahashi^2^, Tomoya Uehara^1^*

List of Supplementary Materials

Synthesis methods of ligands

**Figure S1.** RP-HPLC chromatograms of [^125^I]I-NpG-L-PSMA, [^125^I]I-NpG-D-PSMA, and [^211^At]At-NpG-PSMA.

**Figure S2.** *In vitro* inhibition curves of [^125^I]DCIT bound to LNCaP cells by I-NpG-L-PSMA and I-NpG-D-PSMA.

**Figure S3.** Structure of [^125^I]I-NpG-PSMA.

**Figure S4.** Structure of [^123^I]GLCE.

**Table S1.** Biodistribution of radioactivity in normal mice after injection of [^125^I]I-NpG-L-PSMA, [^125^I]I-NpG-D-PSMA, and [^67^Ga]Ga-PSMA-617.

**Table S2.** Biodistribution of radioactivity in tumor bearing mice after injection of [^125^I]I-NpG-L-PSMA and [^125^I]I-NpG-D-PSMA.

**Table S3.** Biodistribution of radioactivity in normal mice after injection of [^125^I]I-NpG-PSMA.

**Purity checks and characterization of the compound 8 and NpG conjugated PSMA derivatives.**

**General.** [^125^I]NaI (ca. 3.7 M Bq/µL in 0.01 M NaOH) was purchased from Perkin Elmer (Waltman, MA). ^211^At was produced by the ^209^Bi(α, 2n)^211^At reaction followed by separation and purification by a dry distillation method in Fukushima Medical University. ^1^H-NMR and ^13^C-NMR spectra were obtained by a JEOLJNM-ECS-400 spectrometer (JEOL, Tokyo, Japan). Mass spectrometry was carried out using an AccuTOF LC-plus (JMS-T100LP, JEOL). HPLC analysis and purification were performed using a Hitachi L-2400 system coupled to UV detector and a NaI(Tl) radioactivity detector (Gabi star, Raytest, Strubenhardt, Germany). Analytical reversed-HPLC (RP-HPLC) was performed with a Cadenza CD-C18 column (4.6 × 150 mm, Imtakt, Kyoto, Japan) at a flow rate of 1 mL/min using a linear gradient mobile phase starting from 90% A (A: 0.1% aqueous trifluoroacetic acid (TFA)) and 10% B (B: 0.1% TFA in acetonitrile) to 60% A and 40% B in 30 min, followed by 0% A and 100% B in 40 min (System 1), from 80% A and 20% B to 60% A and 40% B in 30 min (System 2), or from 25% C (C: Milli-Q Water) and 75% D (D: acetonitrile) to 0% C and 100% D in 25 min, followed by 0% A and 100% B in 30 min (System 3). Preparative RP-HPLC was performed with a Cadenza CD-C18 column (20 × 150 mm, Imtact) connected with a Cadenza CD-C18 guard column (20 × 20 mm, Imtact) at flow rate of 5 mL/min using a linear gradient mobile phase starting from 20% A and 80% B to 50% A and 50% B in 30 min, followed by a change to 0% A and 100% B at 40 min (System 4). Sep-Pak purification was performed as follows. The cartridge was activated with acetonitrile (5 mL) and water (5 mL), then loaded a sample. The cartridge was washed with water (5 mL) and eluted with acetonitrile (3 mL). *N*-[*N*-[(*S*)-1,3-dicarboxypropyl]carbamoyl]-*S*-3-[^125^I]iodo-L-tyrosine ([^125^I]DCIT) and H-Lys(OtBu)-CO-Glu(OtBu)-OtBu (compound **2**) were synthesized according to the procedures as described before[1]. Other reagents were of analytical grade and used as received.

**Synthesis of Fmoc-D-Glu(OtBu)-D-Glu(OtBu)-2-Nal-OH (1).** Fmoc-D-Glu(OtBu)-D-Glu(OtBu)-2-Nal-OH was synthesized by manual Fmoc solid-phase peptide synthesis using Cl-Trt(2-Cl)-Resin (418 mg, 0.56 mmol) as the solid phase and the following protected amino acids , Fmoc-D-Glu(OtBu) and Fmoc-2-Nal-OH. The peptide chain was constructed manually according to the method consisting of 2 h of coupling of the protected amino acid derivative (2.5 eq.) in the presence of *N, N’*-diisopropylcarbodiimide (DIC, 2.5 eq.) and 1-hydroxybenzotriazol/H_2_O (HOBt, 2.5 eq.) in *N,N’*-dimethylformamide (DMF, 3 mL). After constructing the peptide sequence, the peptide resin was treated with a mixture of acetic acid/2,2,2-trifluoroethanol/dichloromethane (3/1/6; 5 mL) for 2 h to cleave the assembled peptide from the resin. After removing the solvent *in vacuo*, the residue was purified by column chromatography on silica gel eluted with a mixture of chloroform and methanol to afford compound **1** as a white solid (306.8 mg, 0.380 mmol, 86.6%). ^1^H NMR (CDCl_3_) δ: 1.38 (s, 9H, C*H_3_*), 1.42 (s, 9H, C*H_3_*), 1.61-1.86 (m, 4H, C*H_2_*), 1.88-2.03 (m, 2H, C*H_2_*), 2.08-2.33 (m, 4H, C*H_2_* ), 3.15 (q, *J*=1.00 Hz, 1H, C*H_2_*), 3.24-3.43 (m, 1H, C*H_2_*), 4.02 (br. s, 1H, N*H*), 4.10-4.24 (m, 2H, C*H*), 4.30-4.39 (m, 2H, C*H_2_*), 4.50 (m, 1H, C*H*), 4.87 (m, 1H, C*H*), 5.93 (d, *J*=7.32 Hz, 2H, N*H*), 7.28-7.42 (m, 7H, C*H*), 7.51-7.65 (m, 4H, C*H*), 7.74 (m, 4H, C*H*). ESI-MS. m/z [M + Na]^+^ 830, Found 830.

**Synthesis of Fmoc-D-Glu(OtBu)-D-Glu(OtBu)-2-Nal-Lys(OtBu)-CO-Glu(OtBu)-OtBu (3).** Compound **1** (485 mg, 0.60 mmol), compound **2** (292 mg, 0.60 mmol), HOBt (137 mg, 0.90 mmol) and *N,N*-diisopropylethylamine (DIPEA, 153 µL, 0.90 mmol) were dissolved in dichloromethane (5 mL). After cooling on ice, WSCI/HCl dissolved in dichloromethane (1 mL) was added dropwise to the solution. After mixing on ice for 10 min, the reaction solution was stirred at room temperature for 2.5 h. The organic solvent was washed with sat. NaHCO_3_ solution (10 mL), brine (10 mL), 5% citric acid solution (10 mL), and brine (10 mL) successively, and then dried over Na_2_SO_4_. After removing the solvent *in vacuo*, the residue was purified by column chromatography using chloroform and methanol as a solvent to afford compound **3** as a white solid (328 mg, 0.26 mmol, 42.7%). ^1^H NMR (CDCl_3_) δ: 1.24-1.29 (m, 2H, C*H_2_*), 1.40-1.44 (m, 45H, C*H_3_*), 1.58-1.68 (m, 2H, C*H_2_*), 1.70-2.01 (m, 8H, C*H_2_*), 2.08 (dt, *J*=19.78, 6.12 Hz, 4H, C*H_2_*), 2.32 (t, *J*=1.00 Hz, 2H, C*H_2_*), 2.44 (t, *J*=6.17 Hz, 2H, C*H_2_*), 2.73-3.06 (m, 2H, C*H_2_*), 3.15 (dd, *J*=13.72, 8.69 Hz, 2H, C*H_2_*), 4.17-4.25 (m, 3H, C*H*), 4.28-4.44 (m, 4H, C*H_2_,* C*H*), 4.82-4.97 (m, 1H, C*H*), 5.97 (br. s, 2H, N*H*), 6.42-6.65 (m, 1H, N*H*), 7.34-7.43 (m, 5H, C*H*), 7.50-7.66 (m, 5H, C*H*), 7.73 (s, 5H, C*H*). ESI-MS. m/z [M + H]^+^ 1278, Found 1278.

**Synthesis of D-Glu(OtBu)-D-Glu(OtBu)-2-Nal-Lys(OtBu)-CO-Glu(OtBu)-OtBu (4).** Compound **3** (328 mg, 0.26 mmol) was dissolved in 20% piperidine/DMF (10 mL) and then mixed for 30 min. After removing the solvent, the residue was purified by column chromatography using chloroform and methanol as a solvent to afford compound **4** as a white solid (246 mg, 0.23 mmol, 90.6%). ^1^H NMR (CDCl_3_) δ: 1.22-1.28 (m, 2H, C*H_2_*), 1.39-1.43 (m, 45H, C*H_3_*), 1.57 (s, 2H, C*H_2_*), 1.82-1.90 (m, 6H, C*H_2_*), 1.95-2.00 (m, 2H, C*H_2_*), 2.04-2.09 (m, 2H, C*H_2_*), 2.29-2.36 (m, 4H, C*H_2_*), 3.06-3.24 (m, 2H, C*H_2_*), 3.28-3.36 (m, 2H, C*H_2_*), 3.40 (s, 1H, C*H*), 4.12-4.22 (m, 1H, C*H*), 4.26-4.35 (m, 2H, C*H*), 4.83-4.96 (m, 1H, C*H*), 5.76 (m, 1H, N*H*), 5.97-6.11 (m, 1H, N*H*), 7.41-7.45 (m, 2H, C*H*), 7.66-7.80 (m, 5H, C*H*), 7.94 (br. s, 1H, C*H*). ESI-MS. m/z [M + H]^+^ 1056, Found 1056.

**Synthesis of 2-((5-(hydroxymethyl)-2,2-dimetyl-1,3-dioxan-5-yl)methoxy)acetic acid (pro-Neopentyl(OH)-COOH) (6).** Sodium hydride (60%, 58 mg, 1.45 mmol) was washed with hexane and the suspended in tetrahydrofuran (THF, 500 µL) under Ar. Compound **5** (127 mg, 0.72 mmol) dissolved in THF (2 mL) was added dropwise to the suspension on ice. After mixing for 15 min, bromoacetic acid (100 mg, 0.72 mmol) dissolved in THF (500 µL) was added to the suspension at same temperature and the mixed at room temperature overnight. After adding methanol, the solvent was removed *in vacuo*. The residue was purified with preparative TLC using chloroform : methanol (3:1) as a solvent to afford compound **6** as colorless oil (69.2 mg, 0.30 mmol, 41%). ^1^H NMR (CDCl_3_) δ: 1.39 (s, 6H, C*H_3_*), 3.62-3.75 (m, 8H, C*H_2_*), 3.86 (s, 2H, C*H_2_*). ESI-MS. m/z [M + Na]^+^ 257, Found 257.

**Synthesis of pro-Neopentyl(OH)-D-Glu(OtBu)-D-Glu(OtBu)-2-Nal-Lys(OtBu)-CO-Glu(OtBu)-OtBu (7).** Compound **4** (250 mg, 0.237 mmol), compound **6** (55 mg, 0.24 mmol), HOBt (62 mg, 0.41 mmol) was dissolved in chloroform (5 mL) and then DIPEA (65 µL, 0.38 mmol) was added. After colling on ice, WSCI/HCl (96 mg, 0.50 mmol) dissolved in chloroform (1 mL) was added dropwise to the solution. After mixing for 10 min, the reaction mixture was stirred for 2 h at room temperature. The organic layer was washed with 5% citric acid (10 mL) and brine (10 mL × 2), successively and dried over Na_2_SO_4_. After removing the solvent *in vacuo*, the residue was purified with column chromatography using chloroform and methanol as a solvent to afford compound **7** as a white solid (222 mg, 0.175 mmol, 73%). ^1^H NMR (CDCl_3_) δ: 1.22-1.28 (m, 2H, C*H_2_*), 1.39-1.43 (m, 51H, C*H_3_*), 1.53-1.59 (m, 2H, C*H_2_*), 1.82-1.97 (m, 6H, C*H_2_*), 2.05 (dd, *J*=13.95, 7.55 Hz, 4H, C*H_2_*), 2.29-2.37 (m, 4 H), 2.76-2.98 (m, 2H, C*H_2_*), 3.16-3.36 (m, 2H, C*H_2_*), 3.43-3.53 (m, 1H, C*H_2_*), 3.61 (s, 2H, C*H_2_*), 3.64-3.73 (m, 6H, C*H_2_*), 4.03 (s, 1H, O*H*), 4.19-4.24 (m, 1H, C*H*), 4.26-4.32 (m, 2H, C*H*), 4.37-4.45 (m, 1H, C*H*), 4.67-4.87 (m, 1H, C*H*), 5.85 (d, *J*=7.78 Hz, 1H, N*H*), 7.41-7.44 (m, 2H, C*H*), 7.65-7.67 (m, 1H, C*H*), 7.75 (br. s, 3H, C*H*), 8.00 (d, *J*=7.32 Hz, 1H, C*H*). ESI-MS. m/z [M + Na]^+^ 1294, Found 1294.

**Synthesis of pro-Neopentyl(OTf)-D-Glu(OtBu)-D-Glu(OtBu)-2-Nal-Lys(OtBu)-CO-Glu(OtBu)-OtBu (8).** Compound **7** (51 mg, 40 µmol) was dissolved in dry dichloromethane (500 µL) and then 2,6-lutidine (137 µL, 1.18 mmol) was added. After cooling to -78 ˚C, trifluoromethanesulfonic anhydride (32 µL, 198 µmol) dissolved in dry dichloromethane (50 µL) was added dropwise to the solution at -78 ˚C. After mixing at -78 ˚C for 1 h, the solution was mixed at -20 ˚C overnight. After mixing at room temperature for 30 min, the organic layer was washed with sat. NaHCO_3_ solution (5 mL × 2), 5% citric acid solution (5 mL × 2), and brine (15 mL), successively, and then dried over Na_2_SO_4_. After removing the solvent *in vacuo*, the residue was purified with preparative TLC using hexane : ethyl acetate (1:2) as a solvent to afford compound **8** as a white solid (21.4 mg, 15.2 µmol, 38%). ^1^H NMR (CDCl_3_) δ: 1.25 (br. s, 2H, C*H_2_*), 1.33-1.62 (m, 51H, C*H_3_*), 1.72 (br. s, 2H, C*H_2_*), 1.90-2.17 (m,0 8H, C*H_2_*), 2.27-2.43 (m, 4H, C*H_2_*), 2.76-3.05 (m, 2H, C*H_2_*), 3.14-3.36 (m, 2H, C*H_2_*), 3.72-4.08 (m, 8H, C*H_2_*), 4.22-4.49 (m, 4H, C*H*), 4.79 (br. s, 1H, C*H*), 5.79-5.96 (m, 1H, N*H*), 7.40 - 7.54 (m, 3H, C*H*), 7.64-7.80 (m, 4H, C*H*). ESI-MS. m/z [M + Na]^+^ 1426, Found 1426.

**Synthesis of pro-Neopentyl(I)-D-Glu(OtBu)-D-Glu(OtBu)-2-Nal-Lys(OtBu)-CO-Glu(OtBu)-OtBu (9).** Compound **8** (20 mg, 14.8 µmol) and sodium iodide (22 mg, 148 µmol) was dissolved in acetonitrile (1 mL) and then mixed for 100 ˚C for 6 h. After removing the solvent in vacuo, the residue was dissolved in ethyl acetate (5 mL). The organic layer was washed with sat. sodium thiosulfate solution (5 mL× 2) and brine (5 mL× 2) and then dried over Na_2_SO_4_. After removing the solvent *in vacuo*, the residue was purified with a preparative TLC using chloroform : methanol (15 : 1) to afford compound **8** as a white solid (10 mg, 7.24 µmol, 48.8%).^1^H NMR (CDCl_3_) δ: 1.23 (overlapped, 2H, C*H_2_*), 1.30 (s, 6H, C*H_3_*), 1.37-1.42 (d, 45H, C*H_3_*), 1.51-2.39 (m, 16H, C*H_2_*), 3.09-3.13 (m, 2H, NC*H_2_*), 3.35 (s, 2H, I-C*H_2_*), 3.57-3.81 (m, 8H, C*H_2_*), 4.00 (s, 2H, OC*H_2_*), 4.28-4.29 (m, 2H, C*H*), 4.84-4.88 (t, 1H, C*H*), 7.36-7.74 (m, 7H, aromatic). ESI-MS. m/z [M + Na]^+^ 1404, Found 1404.

**Synthesis of Neopentyl(I)-D-Glu-D-Glu-2-Nal-Lys-CO-Glu-OH (I-NpG-D-PSMA).** Compound **9** (10 mg, 7.24 µmol) was dissolved in the mixture of TFA (900 µL) and H_2_O (100 µL) and mixed overnight. The solvent was removed with N_2_ gas and neutralized with 1 N NaOH to pH 7. The solution was purified with RP-HPLC (system 4) to afford **I-NpG-D-PSMA** as a white solid (3.1 mg, 2.92 µmol, 40.4%). ^1^H NMR (D_2_O) δ: 0.98-1.02 (m, 2H, C*H_2_*), 1.21-1.23 (m, 2H, C*H_2_*), 1.42-1.49 (m, 2H, C*H_2_*), 1.72-2.41 (m, 12H, C*H_2_*), 2.96-3.01 (m, 2H, NC*H_2_*), 3.21-3.25 (t, 1H, C*H*), 3.29-3.52 (m, 10H, C*H_2_*), 4.02 (s, 2H, OC*H_2_*), 4.22-4.26 (m, 2H, C*H*), 4.61-4.63 (t, 1H, C*H*), 7.38-7.86 (m, 7H, aromatic). ESI-MS. m/z [M - H]^-^ 1059, Found 1059.

**Synthesis of pro-Neopentyl(OMs)-L-Glu(OtBu)-L-Glu(OtBu)-2-Nal-Lys(OtBu)-CO-Glu(OtBu)-OtBu (9).** L-form of compound **9** was synthesized in the same manner as the synthetic procedure of D-form of compound **9** using Fmoc-L-Glu(OtBu) and methansulfonyl chloride instead of Fmoc-L-Glu(OtBu) and trifluoromethanesulfonic anhydride. ESI-MS. m/z [M + Na]+ 1372, Found 1372.

**Synthesis of Neopentyl(I)-L-Glu-L-Glu-2-Nal-Lys-CO-Glu-OH (I-NpG-L-PSMA).** I-NpG-L-PSMA was synthesized in the same manner as the synthetic procedure of I-NpG-PSMA using L-form of compound **9** instead of D-form of compound **9**. ESI-MS. m/z [M - H]^-^ 1059, Found 1059.

**Preparation of [^125^I]I-NpG-D-PSMA.** Compound **8** (100 µg) was dissolved in 1% diisopropylethylamine (DIPEA)/acetonitrile (25 µL) and then [^125^I]NaI (1.0 µL) was added. After incubated at 90 ˚C for 15 min, the solvent was diluted with H_2_O (100 µL) and then purified with Sep-Pak. After removing the solvent *in vacuo*, the residue was dissolved in the mixture of TFA and water (9 : 1, 300 µL) and stirred for 30 min. The solvent was concentrated by N_2_ gas and neutralized with 1 M NaOH to about pH 4. The product was purified by RP-HPLC (system 1) to afford [^125^I]I-NpG-D-PSMA. After removing the TFA with Sep-Pak, [^125^I]I-NpG-PSMA was diluted in PBS and used for further experiments.

**Preparation of [^125^I]I-NpG-L-PSMA.** [^125^I]I-NpG-L-PSMA was synthesized in the same manner as the synthetic procedure of [^125^I]I-NpG-D-PSMA using L-form of compound **8** instead of D-form of compound **8**.

**Preparation of [^211^At]At-NpG-D-PSMA.** Compound **8** (100 µg) was dissolved in 1% DIPEA/acetonitrile (25 µL) and then ^211^At in acetonitrile (10 µL) was added. After incubated at 90 ˚C for 15 min, the solvent was diluted with H_2_O (100 µL) and then purified with Sep-Pak. After removing the solvent *in vacuo*, the residue was dissolved in the mixture of TFA and water (9 : 1, 300 µL) and stirred for 20 min. The solvent was concentrated by N_2_ gas and neutralized with 1 M NaOH to about pH 4. The product was purified by RP-HPLC (system 2) to afford [^211^At]At-NpG-D-PSMA. After removing the TFA with Sep-Pak, [^211^At]At-NpG-D-PSMA was diluted in PBS and used for further experiments.

**Synthesis of I-NpG-PSMA.** I-NpG-PSMA was synthesized in the same manner as the synthetic procedure of I-NpG-D-PSMA without D-glutamic acid linker. ^1^H NMR (D_2_O) δ: 1.27-1.29 (m, 2H, C*H_2_*), 1.47-1.56 (m, 2H, C*H_2_*), 1.87-1.89 (m, 2H, C*H_2_*), 2.02-2.07 (m, 2H, C*H_2_*), 2.40-2.43 (m, 2H, COC*H_2_*), 2.89-2.91 (t, 2H, ArC*H_2_*), 3.05-3.31 (m, 11H, *neopentyl*, NC*H_2,_ CH*), 3.92-3.96 (s, 2H, OC*H_2_*), 4.15-4.16 (m, 2H, C*H*), 7.38-7.90 (m, 7H, aromatic). ESI-MS. m/z [M - H]^-^ 801, Found 801.

**Preparation of [^125^I]I-NpG-PSMA.** [^125^I]I-NpG-PSMA was synthesized in the same manner as the synthetic procedure of [^125^I]I-NpG-D-PSMA without D-glutamic acid linker.

**Lipophilicity (log *D*_7.4_) measurement**

[^125^I]I-NpG-D-PSMA (3.7 kBq) was mixed with equal amounts (3.0 mL) of 1-octanol and PBS buffer (pH 7.4). The mixture was vortexed for 1 min and allowed to stand for 1 min. After repeating the procedure five times, the mixture was centrifuged at 1,500 g (3740, Kubota, Tokyo) for 10 min. Samples (0.2-2 mL) were taken from each phase and their radioactivity was measured with an auto-well γ-counter. The partition coefficient was determined by calculating the ratio of the counts per minute in the 1-octanol phase to that in the buffer phase and expressed as common logarithm. The results represent the mean of three measurements.

Figure S1.

**Fig. S1.** RP-HPLC chromatograms of [^125^I]I-NpG-L-PSMA, [^125^I]I-NpG-D-PSMA and [^211^At]At-NpG-D-PSMA. The retention times of radiolabeled compounds were identical to those of non-radioactive compounds. In case of [^211^At]At-NpG-D-PSMA, non-radioactive iodine-labeled compound was used.

Figure S2

Fig. S2. *In vitro* inhibition curves of [^125^I]DCIT bound to LNCaP cells by I-NpG-L-PSMA and I-NpG-D-PSMA.

Figure S3

Fig. S3. Structure of [^125^I]I-NpG-PSMA.

Figure S4

Fig. S4. Structure of [^123^I]GLCE.

**Table S1.** Biodistribution of radioactivity in normal mice after injection of [^125^I]I-NpG-L-PSMA, [^125^I]I-NpG-PSMA, and [^67^Ga]Ga-PSMA-617.

| Tissue radioactivity is expressed as %ID/g [for each group, n=3-5; results are reported as mean ± SD.] | | | | |
| --- | --- | --- | --- | --- |
|  | [^125^I]I-NpG-L-PSMA | | | |
| Tissues | 10 min | 1 h | 3 h | 6 h |
| Blood | 1.48 ± 0.20^b^ | 0.26 ±0.01 | 0.04 ± 0.01 | 0.03 ± 0.02 |
| Liver | 1.64 ± 0.33^a^ | 0.99 ± 0.14^ab^ | 0.43 ± 0.01^ab^ | 0.53 ±0.02^ab^ |
| Spleen | 2.23 ± 1.10 | 1.05 ± 0.19^ab^ | 0.22 ± 0.07^ab^ | 0.11 ± 0.03^a^ |
| Kidney | 92.3 ± 11.0^b^ | 113.6 ±3.86^ab^ | 38.6 ± 11.9^a^ | 21.2 ± 9.70^a^ |
| Pancreas | 0.80 ± 0.16 | 1.40 ± 1.78 | 0.12 ± 0.03 | 0.07 ± 0.04 |
| Heart | 1.12 ± 0.24 | 0.60 ± 0.37 | 0.06 ± 0.01 | 0.05 ± 0.05 |
| Lung | 2.15 ± 0.49^a^ | 0.69 ± 0.16^ab^ | 0.21 ± 0.03^ab^ | 0.09 ± 0.03^a^ |
| Muscle | 0.62 ± 0.18^a^ | 0.14 ± 0.03 | 0.10 ± 0.05 | 0.05 ± 0.01 |
| Bone | 0.68 ± 0.18^a^ | 0.36 ± 0.43 | 0.09 ± 0.04 | 0.05 ± 0.01 |
| Intestine* | 2.49 ± 0.66 | 3.40 ± 0.86^a^ | 4.96 ± 0.17^ab^ | 4.60 ± 2.56 |
| Stomach* | 0.33 ± 0.09 | 0.35 ± 0.30 | 0.08 ± 0.02^b^ | 0.23 ± 0.29 |
| Neck* | 0.34 ± 0.09^a^ | 0.07 ± 0.03^a^ | 0.01 ± 0.00 | 0.02 ± 0.01 |
| Urine* |  |  |  | 53.3 ± 5.56 |
| Feces* |  |  |  | 0.45 ± 0.48 |
|  | | | | |
|  | [^125^I]I-NpG-D-PSMA | | | |
| Tissues | 10 min | 1 h | 3 h | 6 h |
| Blood | 1.31 ± 0.10^c^ | 0.18 ±0.02 | 0.04 ± 0.02 | 0.03 ± 0.01 |
| Liver | 0.83 ± 0.12^c^ | 0.17 ± 0.07 | 0.05 ± 0.03 | 0.05 ±0.03 |
| Spleen | 2.09 ± 0.83 | 0.20 ± 0.01 | 0.07 ± 0.05 | 0.05 ± 0.05 |
| Kidney | 58.5 ± 2.27^c^ | 74.6 ± 14.9^c^ | 22.9 ± 16.5 | 17.5 ± 5.43^c^ |
| Pancreas | 0.63 ± 0.15^c^ | 0.02 ± 0.09 | 0.20 ± 0.31 | 0.09 ± 0.12 |
| Heart | 0.76 ± 0.08^c^ | 0.14 ± 0.7 | 0.05 ± 0.04 | 0.03 ± 0.01 |
| Lung | 2.11 ± 0.16^c^ | 0.38 ± 0.06 | 0.12 ± 0.05 | 0.08 ± 0.00^c^ |
| Muscle | 0.47 ± 0.07^c^ | 0.11 ± 0.08 | 0.05 ± 0.03 | 0.06 ± 0.07 |
| Bone | 0.71 ± 0.07^c^ | 0.30 ± 0.25 | 0.21 ± 0.28 | 0.16 ± 0.17 |
| Intestine* | 2.76 ± 0.02^c^ | 2.97 ± 0.24^c^ | 3.30 ± 0.08^c^ | 6.19 ± 4.27 |
| Stomach* | 0.28 ± 0.06 | 0.18 ± 0.00 | 0.27 ± 0.10^c^ | 0.86 ± 0.65 |
| Neck* | 0.48 ± 0.06^c^ | 0.11 ± 0.03^c^ | 0.03 ± 0.02^c^ | 0.07 ± 0.09 |
| Urine* |  |  |  | 30.7 ± 2.85^c^ |
| Feces* |  |  |  | 0.66 ± 0.50 |
|  | | | | |
|  | [^67^Ga]Ga-PSMA-617 | | | |
| Tissues | 10 min | 1 h | 3 h | 6 h |
| Blood | 3.84 ± 0.55 | 0.26 ±0.15 | 0.01 ± 0.00 | 0.01 ± 0.00 |
| Liver | 0.98 ± 0.13 | 0.15 ± 0.03 | 0.06 ± 0.00 | 0.05 ±0.01 |
| Spleen | 1.76 ± 0.68 | 0.12 ± 0.08 | 0.01 ± 0.01 | 0.00 ± 0.00 |
| Kidney | 82.9 ± 12.2 | 11.1 ± 3.22 | 1.12 ± 0.34 | 1.06 ± 0.42 |
| Pancreas | 1.20 ± 0.22 | 0.11 ± 0.04 | 0.00 ± 0.00 | 0.00 ± 0.00 |
| Heart | 1.51 ± 0.13 | 0.10 ± 0.10 | 0.02 ± 0.03 | 0.00 ± 0.00 |
| Lung | 3.52 ± 0.29 | 0.26 ± 0.12 | 0.03 ± 0.02 | 0.02 ± 0.02 |
| Muscle | 1.34 ± 0.18 | 0.15 ± 0.07 | 0.02 ± 0.02 | 0.02 ± 0.02 |
| Bone | 1.54 ± 0.19 | 0.20 ± 0.09 | 0.00 ± 0.00 | 0.00 ± 0.00 |
| Intestine* | 1.58 ± 0.17 | 0.83 ± 0.25 | 0.21 ± 0.09 | 0.34 ± 0.17 |
| Stomach* | 0.32 ± 0.03 | 0.57 ± 0.31 | 0.03 ± 0.02 | 0.05 ± 0.03 |
| Neck* | 0.03 ± 0.01 | 0.01 ± 0.01 | 0.00 ± 0.00 | 0.00 ± 0.00 |
| Urine* |  |  |  | 66.7 ± 15.8 |
| Feces* |  |  |  | 0.19 ± 0.12 |

* Tissue radioactivity was expressed as %ID.

Significance determined by one-way analysis of variance followed by Tukey’s multiple-comparison test.

^a^ *p*<0.05: [^125^I]I-NpG-L-PSMA vs [^67^Ga]Ga-PSMA-617

^b^ *p*<0.05: [^125^I]I-NpG-L-PSMA vs [^125^I]I-NpG-D-PSMA

^c^ *p*<0.05: [^125^I]I-NpG-D-PSMA vs [^67^Ga]Ga-PSMA-617

**Table S2.** Biodistribution of radioactivity in tumor bearing mice after injection of [^125^I]I-NpG-L-PSMA and [^125^I]I-NpG-D-PSMA.

| Tissue radioactivity is expressed as %ID/g [for each group, n=3-5; results are reported as mean ± SD.] | | | | |
| --- | --- | --- | --- | --- |
|  | [^125^I]I-NpG-L-PSMA | | [^125^I]I-NpG-D-PSMA | |
| Tissues | 1 h | 6 h | 1 h | 6 h |
| Blood | 0.66 ± 0.18 | 0.11 ± 0.03^a^ | 0.64 ± 0.04 | 0.06 ± 0.03 |
| Liver | 1.85 ± 0.50^a^ | 0.44 ± 0.02^a^ | 0.79 ± 0.06 | 0.07 ± 0.03 |
| Spleen | 37.0 ± 10.4 | 4.55 ± 1.18^a^ | 30.5 ± 7.49 | 1.55 ± 0.75 |
| Kidney | 223 ± 5.91^a^ | 226 ± 30.0 | 275 ± 17.6 | 160 ± 89.9 |
| Pancreas | 1.17 ± 0.38 | 0.26 ± 0.08^a^ | 0.84 ± 0.25 | 0.12 ± 0.05 |
| Heart | 1.62 ± 0.87 | 0.22 ± 0.06^a^ | 0.89 ± 0.09 | 0.10 ± 0.04 |
| Lung | 2.78 ± 0.93 | 0.59 ± 0.12^a^ | 2.80 ± 0.09 | 0.22 ± 0.09 |
| Muscle | 0.64 ± 0.21^a^ | 0.27 ± 0.31 | 0.27 ± 0.05 | 0.07 ± 0.04 |
| Bone | 0.47 ± 0.12^a^ | 0.09 ± 0.02 | 0.25 ± 0.02 | 0.07 ± 0.02 |
| Intestine* | 2.52 ± 0.29^a^ | 1.87 ± 0.41 | 1.66 ± 0.29 | 1.51 ± 0.61 |
| Stomach* | 0.23 ± 0.04 | 0.15 ± 0.07 | 0.19 ± 0.04 | 0.17 ± 0.10 |
| Neck* | 0.45 ± 0.13^a^ | 3.19 ± 1.28 | 0.77 ± 0.17 | 0.10 ± 0.01 |
| Tumor | 9.19 ± 1.89^a^ | 11.0 ± 2.68^a^ | 20.4 ± 6.92 | 20.8 ± 6.84 |

*: Tissue radioactivity was expressed as %ID.

Significance determined by one-way analysis of variance followed by Tukey’s multiple-comparison test.

^a^ *p*<0.05: [^125^I]I-NpG-L-PSMA vs [^125^I]I-NpG-D-PSMA

**Table S3.** Biodistribution of radioactivity in normal mice after injection of [^125^I]I-NpG-PSMA.

| Tissue radioactivity is expressed as %ID/g [for each group, n=3-5; results are reported as mean ± SD.] | |
| --- | --- |
| Tissues | 1 h |
| Blood | 0.11 ±0.02 |
| Liver | 3.72 ± 2.93 |
| Spleen | 0.33 ± 0.29 |
| Kidney | 25.6 ± 7.08 |
| Pancreas | 0.13 ± 0.04 |
| Heart | 0.08 ± 0.03 |
| Lung | 0.29 ± 0.08 |
| Muscle | 0.14 ± 0.07 |
| Intestine* | 69.7 ± 11.0 |
| Stomach* | 0.26 ± 0.05 |
| Neck* | 0.05 ± 0.02 |

* Tissue radioactivity was expressed as %ID.

(a)

(b)

(c)


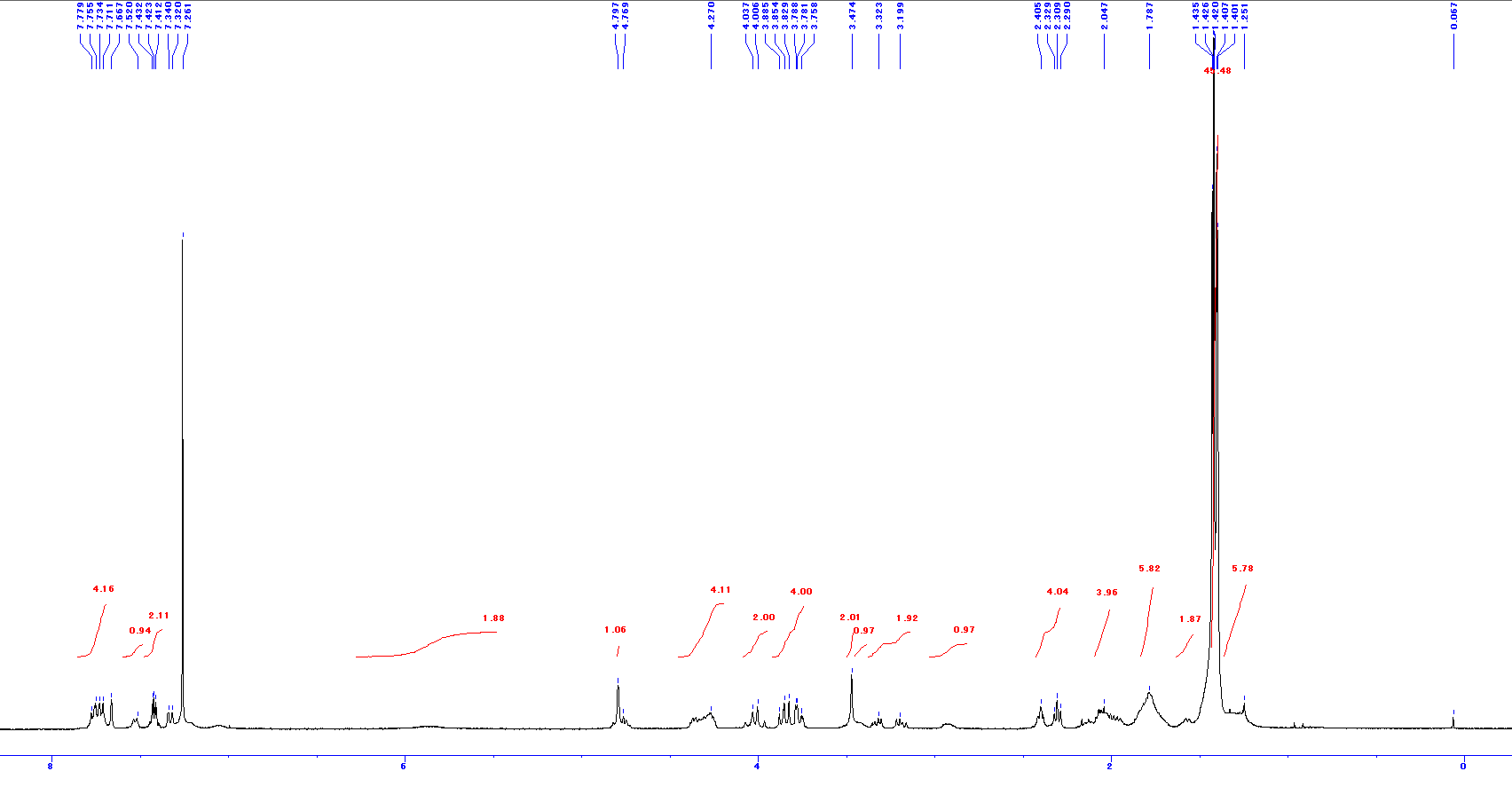


The compound **8**. (a) The chemical structure. (b) HPLC chromatogram (system 3). (c) ^1^H NMR chart.

(a)

(b)

(c)


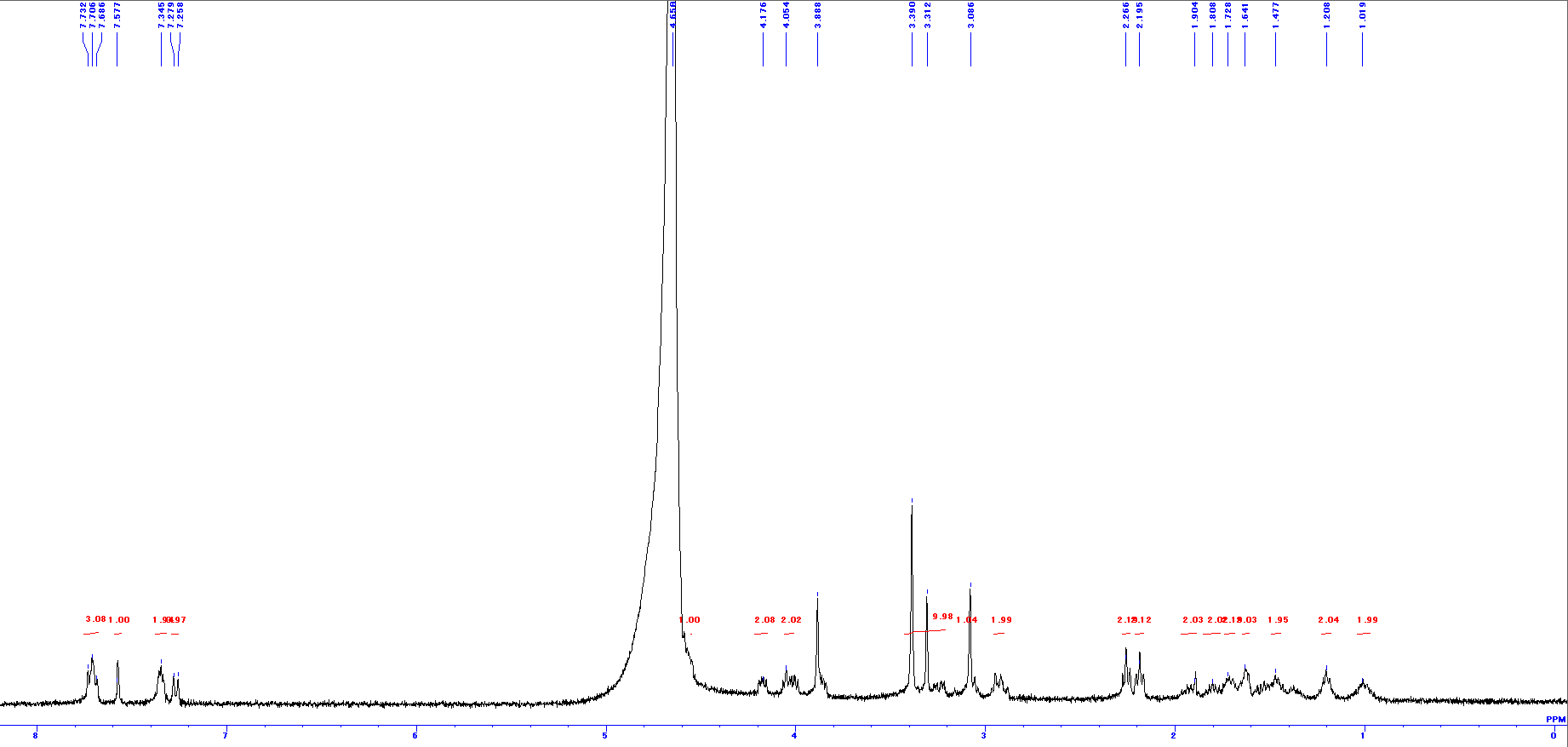


I-NpG-PSMA. (a) The chemical structure. (b) HPLC chromatogram (system 2). (c) ^1^H NMR chart.

Reference

1. Kozikowski AP, Zhang J, Nan F, Petukhov PA, Grajkowska E, Wroblewski JT, et al. Synthesis of urea-based inhibitors as active site probes of glutamate carboxypeptidase II: efficacy as analgesic agents. J Med Chem. 2004;47:1729-38. doi:10.1021/jm0306226.
